# Challenges facing the elimination of sleeping sickness in west and central Africa: sustainable control of animal trypanosomiasis as an indispensable approach to achieve the goal

**DOI:** 10.1186/s13071-015-1254-y

**Published:** 2015-12-16

**Authors:** Gustave Simo, Jean Baptiste Rayaisse

**Affiliations:** Department of Biochemistry, Molecular Parasitology and Entomology Unit, Faculty of Science, University of Dschang, PO Box 67 Dschang, Cameroon; Tsetse ecology and control; CIRDES, 01 BP 454, Bobo-Dioulasso, 01 Burkina Faso

**Keywords:** Sleeping sickness, Animal African trypanosomiasis, Vector control, Disease elimination

## Abstract

African trypanosomiases are infectious diseases caused by trypanosomes. African animal trypanosomiasis (AAT) remains an important threat for livestock production in some affected areas whereas human African trypanosomiasis (HAT) is targeted for elimination in 2020. In West and Central Africa, it has been shown that the parasites causing these diseases can coexist in the same tsetse fly or the same animal. In such complex settings, the control of these diseases must be put in the general context of trypanosomiasis control or “one health” concept where the coordination of control operations will be beneficial for both diseases. In this context, implementing control activities on AAT will help to sustain HAT control. It will also have a positive impact on animal health and economic development of the regions. The training of inhabitants on how to implement and sustain vector control tools will enable a long-term sustainability of control operations that will lead to the elimination of HAT and AAT.

## Letter to editor

Trypanosomiases are infectious diseases caused by trypanosomes, mainly transmitted by tsetse flies. African Animal trypanosomiasis (AAT), one of the most important diseases of livestock, causes major constraints to livestock production in sub-Saharan Africa. As a consequence of these infections, about 34 % of livestock keepers must subsist on less than 1.24 USD per day [1]. In regions of extensive livestock production, control measures have been implemented to reduce the disease burden. However, the epidemiological status of AAT in HAT foci of the forest regions where animal breeding is practiced at small scale is not well understood.

Human African Trypanosomiasis (HAT) is a neglected tropical disease that remains an important public health problem in sub-Saharan Africa. It has been included into the WHO “roadmap for elimination” by 2020. However, the very low prevalence of HAT has led to three types of demotivations that do not plead for this elimination: (1) inhabitants’ demotivation due to their repetitive participations to medical surveys without being infected; (2) technicians’ demotivation due to thousands of people examined without patients, and (3) donors’ demotivation with thousands of US dollars spent for few or no patient(s) detected. To currently achieve the set goals, events that jeopardized the elimination in 1960’ must be avoided. A growing body of evidence indicates that asymptomatic carriers remain after medical surveys [2, 3] and cannot be detected passively [4]. The animal reservoirs of *T. b. gambiense* reported in west [5–9] and central [10–15] Africa are additional factors to be taken into account for the elimination. To achieve elimination of the disease, sustainable control and surveillance measures must be developed to ensure complete interruption of the disease transmission.

## African animal trypanosomiasis in HAT foci of west and central Africa

Various trypanosome species including *T. b. brucei*, *T. congolense*, *T. vivax* and *T. simiae* have been identified in tsetse and animals of HAT foci of west and central Africa. Remarkably, mixed infections consisting of *T. b. gambiense* with other trypanosomes were reported in tsetse and animals. The global infection rate of animal trypanosomes in animals and tsetse flies of HAT foci is generally high and sometime above 70 % (Table 1) [13, 16]. Controlling AAT is becoming urgent, especially, with the prohibition of hunting activities, the socio-economic and environmental mutations observed in most foci and the stimulation of inhabitants to practice animal breeding. In HAT foci where control activities on HAT is ongoing, it is important to see how AAT control will not jeopardize HAT control, and how associated control measures could help to achieve the elimination of these diseases.

**Table 1:** Prevalence of animal trypanosomes in some HAT foci of West and Central Africa

HAT focus	Countries	Prevalence of animal trypanosomes	References
Nditam	Cameroon	24.5 %^b^	[23]
Bipindi	Cameroon	62.6 %^c^; 21 %^b^	[23–26]
Campo	Cameroon	55.7 %^c^; 11.7 %^b^	[23, 25, 26]
Doume	Cameroon	12.5 %^c^; 10 %^b^	[23, 25]
Fontem	Cameroon	73.7 %^c^	[13]
Bafia	Cameroon	25.5 %^a^	[27]
Malanga	DRC	22.4 %^a^	[28]
Mandoul	Chad	22.9 %^c^	[29]
Luba	Equatorial Guinea	28.6 %^a^	[15]
Mbini	Equatorial Guinea	52.6 %^c^	[14]
Kogo	Equatorial Guinea	36.1 %^c^	[14]
Bonon	Côte d’Ivoire	72 %^c^; 28 %^a^,	[16]
Sinfra	Côte d’Ivoire	14 %^a^	[30]

^a^Trypanosomes’ prevalence reported in tsetse flies

^b^Trypanosomes’ prevalence reported in wild animals

^c^Trypanosomes’ prevalence reported in domestic animals

## Need to support AAT control for the elimination of HAT in west and central Africa

In *T. b. gambiense* foci, only human health professionals are involved in HAT control. With the asymptomatic patients and animal reservoirs that remain in these foci after medical surveys, the unilateral activities of health professionals cannot enable sustainable control which is necessary to achieve HAT elimination. In HAT foci, the data generated on AAT plead for its control, at least, to improve animal health and peasant economy. Controlling AAT will be beneficial for HAT because the two diseases can be transmitted by the same tsetse and the parasites causing these diseases are often found in the same tsetse fly or the same animal. In such context, HAT control must be put in the general context of trypanosomiasis control or “One health” where strategies put in place must ensure both the control of HAT and AAT, and where AAT control will generate real societal benefits on human health than a single action on HAT. To achieve the elimination or interrupt trypanosomes’ transmission, medical surveys must be associated to the treatment of animals and also vector control that can ensure the elimination of infected tsetse as well as flies susceptible to get infection on asymptomatic carriers or infected animals. With new tools developed recently, vector control costs have been considerably reduced [17, 18] and their implementation has been very promising in some foci [19]; showing that money is being put in HAT control, both by medical surveys and tsetse control. In foci where tsetse control is being done to stop HAT transmission and where animals are bred, there is also, and already, an impact on AAT transmission. Once the tsetse flies would have been killed, the transmission of human and animal trypanosomiases could not occur anymore. Hence, for just “a few dollars more” than those spent already, significant impacts on AAT are also expected in areas where both HAT and AAT coexist and animal trypanosomiases would no longer be a problem in such areas. The challenge faced by the sustainability of vector control can be overcome by integrating control activities of both diseases. The sensitization of inhabitants on the pathological impacts of AAT on animal health and peasant economy will ease their cooperation for control activities that will guarantee and ensure sustainability and success of control measures. Current data on AAT are useful arguments to sensitize inhabitants and Governments on the threats and impacts of AAT on peasant economy in HAT foci. Showing the need of long term control of AAT for animal and human health, actors of different ministries could cooperate and join their efforts to achieve the elimination of these diseases. Improvement of trypanosomiases control in HAT foci requires close cooperation between technical structures in charge of AAT and HAT control. This is a critical step for the maintenance of sustainable control activities that will provide mutual benefits for human and animal health and could lead to the elimination of African trypanosomiases. Monitoring of trypanosome prevalence in animals and/or tsetse, as well as human exposure to tsetse bites [19–22] could constitute interesting and reliable indicators to evaluate the success of control operations.

## Conclusion

HAT control can be sustained through AAT control; by putting the control of these diseases in the “one health” concept. Implementing control activities on AAT will help to sustain HAT control and the sensitization of inhabitants on AAT and their training will enable to sustain control operations that will interrupt trypanosomes’ transmission.

## References

[1] Perry B, Sones K (2007). Poverty reduction through animal health. Science..

[2] Jamonneau V, Ilboudo H, Kaboré J, Kaba D, Koffi M, Solano P (2012). Untreated human infections by *Trypanosoma brucei gambiense* are not 100 % fatal. PLoS Negl Trop Dis.

[3] Kaboré J, Koffi M, Bucheton B, MacLeod A, Duffy C, Ilboudo H (2011). First evidence that parasite infecting apparent aparasitemic serological suspects in human African trypanosomiasis are *Trypanosoma brucei gambiense* and are similar to those found in patients. Infect Genet Evol.

[4] WHO Control and surveillance of human African trypanosomiasis: report of a WHO expert committee. Geneva, Switzerland: WHO technical reports series. 2013; n°984: 237pp

[5] Molyneux DH (1973). Animal reservoir and gambian trypanosomiasis. Ann Soc Belge Méd Trop..

[6] Molyneux DH (1980). Animal reservoirs and residual foci of *Trypanosoma brucei gambiense* sleeping sickness in West Africa. Insect Sci Appl..

[7] Gibson W, Mehlitz D, Lanham SM, Godfrey DG (1978). The identification of *Trypanosoma brucei gambiense* in Liberian pigs and dogs by isoenzymes and by resistance to human plasma. Tropenmedizin Parasitol..

[8] Guedegbe B, Verhulsta A, Van Meirvennen N, Pandey VS, Doko A (1992). Indications sérologiques de l’existence d’un réservoir sauvage de *Trypanosoma brucei gambiense* dans la réserve de biosphère de la Pendjari en République du Bénin. Ann Soc Belge Méd Trop.

[9] Mehlitz D, Zillmann U, Scott CM, Godfrey DG (1982). Epidemiological studies on the animal reservoir of gambiense sleeping sickness. III. Characterization of *Trypanozoon* stocks by isoenzymes and sensitivity to human serum. Tropenmedizin Parasitol.

[10] Njiokou F, Laveissière C, Simo G, Nkinin S, Grébaut P, Cuny G (2006). Wild fauna as probable animal reservoir for *Trypanosoma brucei gambiense* in Cameroon. Infect Genet Evol..

[11] Njiokou F, Nimpaye H, Simo G, Njitchouang GR, Asonganyi T, Cuny G (2010). Domestic animals as potential reservoir hosts of *Trypanosoma brucei gambiense* in sleeping sickness foci in Cameroon. Parasite..

[12] Nkinin SW, Njiokou F, Penchenier L, Grebaut P, Simo G, Herder S (2002). Characterization of *T. brucei* s.l. by isoenzymes in domestic pigs from the Fontem sleeping sickness focus of Cameroon. Acta Trop.

[13] Simo G, Asonganyi T, Nkinin SW, Njiokou F, Herder S (2006). High prevalence of *Trypanosoma brucei gambiense* group 1 in pigs from the Fontem sleeping sickness focus in Cameroon. Vet Parasitol..

[14] Cordon-Obras C, Berzosa P, Ndong-Mabale N, Bobuakasi L, Buatiche JN, Ndongo-Asumu P (2009). Trypanosoma brucei gambiense in domestic livestock of Kogo and Mbini foci (Equatorial Guinea. Trop Med Int Health.

[15] Cordon-Obras C, García-Estébanez C, Ndong-Mabale N, Abaga S, Ndongo-Asumu P, Benito A (2010). Screening of *Trypanosoma brucei gambiense* in domestic livestock and tsetse flies from an insular endemic focus (Luba, Equatorial Guinea). PLoS Negl Trop Dis.

[16] Jamonneau V, Ravel S, Koffi M, Kaba D, Zeze DG, Ndri L (2004). Mixed infections of trypanosomes in tsetse and pigs and their epidemiological significance in a sleeping sickness focus of Côte d’Ivoire. Parasitology..

[17] Esterhuizen J, Rayaisse JB, Tirados I, Mpiana S, Solano P, Vale GA (2011). Improving the cost effectiveness of visual devices for the control of riverine tsetse flies, the major vectors of human African trypanosomiasis. PLoS Negl Trop Dis..

[18] Rayaisse JB, Esterhuizen J, Tirados I, Kaba D, Salou E, Diarrassouba A (2011). Towards an optimal design of target for tsetse control: comparisons of novel targets for the control of *Palpalis* group tsetse in West Africa. Plos Negl Trop Dis.

[19] Courtin F, Camara M, Rayaisse JB, Kagbadouno M, Dama E, Camara O (2015). Reducing Human-Tsetse Contact Significantly Enhances the Efficacy of Sleeping Sickness Active Screening Campaigns: A Promising Result in the Context of Elimination. PLoS Negl Trop Dis.

[20] Somda MB, Bengaly Z, Dama E, Poinsignon A, Dayo G-K, Sidibe I (2013). First insights into the cattle serological response to tsetse salivary antigens: A promising direct biomarker of exposure to tsetse bites. Vet Parasitol.

[21] Dama E, Cornélie S, Somda MB, Camara M, Kambiré R, Courtin F (2013). Identification of *Glossina palpalis gambiensis* specific salivary antigens: towards the development of a serologic biomarker of human exposure to tsetse flies in West Africa. Microbes Infect..

[22] Dama E, Cornelie S, Camara M, Somda MB, Poinsignon A, Ilboudo H (2013). In Silico Identification of a Candidate Synthetic Peptide (Tsgf118–43) to Monitor Human Exposure to Tsetse Flies in West Africa. PLoS Negl Trop Dis.

[23] Njiokou F, Simo G, Nkinin S, Herder S (2004). Infection rate of *Trypanosoma brucei* s.l., *T. vivax, T. congolense* “forest type” and *T. simiae* in small wild vertebrates in south Cameroon. Acta Trop.

[24] Tchouomene-Labou J, Nana-Djeunga H, Simo G, Njitchouang GR, Cuny G, Asonganyi T (2013). Spatial and temporal variations relevant to tsetse control in the Bipindi focus of southern Cameroon. Parasit Vectors..

[25] Nimpaye H, Njiokou F, Njine T, Njitchouang GR, Cuny G, Herder S (2011). *Trypanosoma vivax*, *T. congolense* « forest type » and *T. simiae*: Prevalence in domestic animals of sleeping sickness foci of Cameroon. Parasite.

[26] Herder S, Simo G, Nkinin S, Njiokou F (2002). Detection of trypanosomes in wild animals from southern Cameroon using Polymerase Chain Reaction (PCR). Parasite..

[27] Simo G, Fongho P, Farikou O, Ndjeuto-Tchouli PI, Tchouomene-Labou J, Njiokou F (2015). Trypanosome infection rates in tsetse flies in the “silent” sleeping sickness focus of Bafia in the Centre Region in Cameroon. Parasit Vectors.

[28] Simo G, Silatsa BA, Njiokou F, Lutumba P, Mansinsa PD, Madinga JN (2012). Identification of different trypanosome species in the mid-guts of tsetse flies of the Malanga (Kimpese) sleeping sickness focus of the Democratic Republic of Congo. Parasit Vectors..

[29] Mallaye P, Kohagne Tongué L, Ndjeleje N, Louis FJ, Mahamat Hassane H (2014). Transmission concomitante de Trypanosomose humaine et animale: le foyer du Mandoul au Tchad. Rév Elev Med Vét Pays Trop.

[30] Masiga DK, McNamara JJ, Laveissière C, Truc P, Gibson WC (1996). A high prevalence of mixed infections in tsetse flies in Sinfra, Côte d’Ivoire, detected by DNA amplification. Parasitology..

